# Atherosclerosis in Vietnamese patients with systemic sclerosis and its relationship to disease and traditional risk factors

**DOI:** 10.1093/rap/rkac048

**Published:** 2022-06-02

**Authors:** Thuy Nguyen Thi Phuong, Trang Dao Thi, Ingrid E Lundberg, Binh Nguyen Huy

**Affiliations:** Rheumatology Department, Bach Mai Hospital; Internal Medicine Department, Hanoi Medical University, Hanoi, Vietnam; Internal Medicine Department, Hanoi Medical University, Hanoi, Vietnam; Division of Rheumatology, Department of Medicine, Solna; Center for Molecular Medicine, Karolinska Institutet; Rheumatology Clinic, Karolinska University Hospital, Stockholm, Sweden; Physiology Department, Hanoi Medical University, Hanoi, Vietnam

**Keywords:** SSc, atherosclerosis, carotid intima–media thickness, lipid profile

## Abstract

**Objective:**

The aim of this study was to determine the frequency of clinical and subclinical atherosclerosis in Vietnamese patients with SSc and the risk factors for subclinical atherosclerosis.

**Methods:**

A case–control study of 46 patients with SSc who met the ACR criteria for the disease and 42 healthy age- and sex-matched controls of Kinh ethnicity was conducted. Clinical data including cardiovascular disease (CVD) events were collected. Serum levels of blood lipids and high-sensitivity CRP were determined. Carotid artery intima–media thickness (IMT) and carotid plaques were measured by carotid Doppler ultrasonography.

**Results:**

Patients with SSc, of whom 96% had dcSSc, reported a higher number of CVD events compared with the controls (21.7 *vs* 0%; *P* = 0.0065). They exhibited low serum levels of high-density lipoprotein cholesterol and high levels of total cholesterol compared with controls (*P* = 0.01 and *P* = 0.03, respectively). Common carotid artery IMT was significantly higher in SSc patients compared with controls [mean (s.d.): 0.61 (0.12) *vs* 0.47 (0.07) mm; *P* < 0.0001]. Carotid artery IMT in SSc showed significant positive correlations with age, disease duration, total cholesterol and low-density lipoprotein cholesterol (*P* < 0.05). Thirteen patients with SSc (28.3%) but no controls had carotid atherosclerotic plaques. Patients with plaque had a higher mean modified Rodnan skin score and higher mean IMT compared with patients without plaque.

**Conclusion:**

We confirmed an increased risk of CVD events and signs of subclinical atherosclerosis in patients with SSc of Kinh ethnicity and both traditional and disease-related risk factors for CVD.

Key Messages

- SSc, in particular dcSSc, was associated with clinical and subclinical accelerated atherosclerosis.
- dcSSc patients had an increased number of carotid plaques and increased carotid artery intima–media thickness.
- Carotid atherosclerosis in SSc occured at an older age and after a longer disease duration compared with controls.
- SSc patients with carotid plaque had more severe disease as estimated by modified Rodnan skin score.

## Introduction

SSc is a chronic autoimmune disease characterized by endothelial dysfunction, microvascular damage and increased tissue fibrosis. SSc has two main subtypes, namely lcSSc and dcSSc. Both subtypes have an effect on the cardiovascular system. Involvement of the microvasculature is one of the earliest features of SSc, preceding and potentially contributing via tissue ischaemia to the widespread fibrosis characteristic of this condition. The pathogenesis of the vasculopathy is not fully understood, but immune reactions to viral or environmental factors, reperfusion injury or anti-endothelial antibodies might all be involved [1]. Endothelial damage and vascular dysfunction play a central role from early in the pathogenesis of SSc and in the evolution of the internal organ dysfunction. The microvascular abnormalities contribute to the pathogenesis of RP, digital ulcers, pulmonary arterial hypertension and scleroderma renal crisis. Although microvascular disease is the hallmark of SSc, macrovascular disease, with clinical manifestations such as cardiovascular disease (CVD), is also increased in SSc compared with healthy subjects but has been less well characterized [2].

Vietnam is situated in the Southeast Asian region, with a population of ∼98 million. The Kinh (Viet) people account for 85% of the population and live primarily in the urban areas, with CVD being the leading cause of the morbidity and mortality in Vietnam [3]. Previous epidemiological studies have indicated that hypertension is one of the most common causes of CVD, followed by total cholesterol, obesity and smoking in Southeast Asian countries [4, 5]. SSc is a rare disease in Asia, with a pooled prevalence estimate of 6.8 per 100 000 and a pooled incidence rate of 0.9 per 100 000 person-years [6]. Although the scarce data from Vietnam did not allow us to provide an estimate of the incidence and prevalence of SSc in Vietnam, the occurrence of disease in Vietnam is low. From studies published in English and in Vietnamese, the majority of SSc patients in Vietnam are female (>70%) and aged between 48 and 60 years at diagnosis [7, 8]. Heart damage has been noted in 15–49% of Vietnamese patients with SSc and carries the threat of sudden cardiac death [8]. Cardiovascular manifestations were complex, including pericardial effusion, arrhythmias, myocardial dysfunction, heart failure and peripheral arterial disease. The treatment of SSc in Vietnam still faces several challenges, including a lack of well-trained health professionals, inadequate coverage of health insurance, and the finding that adherence to treatment tends to be lower in rural areas than in urban regions owing to patient education. There is a rheumatology network in Vietnamese provinces. However, only some major hospitals in large cities have rheumatology departments caring for patients with SSc. Vietnam is a low- to middle-income country; hence, the cost of SSc treatment is a considerable burden to a patient's family budget. The coverage by health insurance in Vietnam is still relatively low. The patients with SSc must pay by themselves for the remaining cost, which can range between 20 and 100%. Moreover, the cost of transportation from the countryside to the hospital is high, which, not surprisingly, reduces patient adherence.

In several autoimmune diseases, such as RA and SLE, there is an increased prevalence of CVD [9]. This increased risk is often related to accelerated atherosclerosis and is mostly attributed to the interaction between disease-related chronic inflammation and traditional cardiovascular risk factors. Epidemiological studies suggest that CVD is also significant in patients with SSc, and CVD contributes to approximately one-third of the non-SSc-related deaths. Moreover, deaths from cardiovascular causes occur in SSc patients more than a decade earlier than in general population [10]. Nowadays, atherosclerosis is considered an inflammatory disease, and endothelial cell dysfunction is strongly implicated in its pathogenesis, related, in part, to traditional risk factors, such as smoking and dyslipidaemia. Despite the increased mortality rates in SSc, partly attributable to cardiac involvement, the cardiovascular risk factors and the presence of macrovascular disease have been less well explored. Therefore, in this study we aimed to determine the frequency of subclinical atherosclerosis by measuring intima–media thickness (IMT) and plaque of the common carotid artery in patients with SSc of Kinh ethnicity compared with healthy controls. We also aimed to evaluate risk factors for subclinical atherosclerosis in these patients. In addition, we determined the frequency of clinical events of CVD in our cohort.

## Methods

### Study design and patients

This case–control study included 46 consecutive patients with SSc, classified according to the 1980 ACR criteria [11], attending the Rheumatology Department, Bach Mai hospital, Hanoi, Vietnam between August 2018 and July 2019. Patients were eligible if they were >18 years of age. Pregnancy was an exclusion criterion. SSc patients were classified into respective subsets, with either dcSSc (44 patients) or lcSSc disease (2 patients) according to the distribution of skin thickness as defined by LeRoy and Medsger [12]. The disease duration for SSc was defined as the time since the onset of the first SSc-related symptom other than RP. Forty-two age- and sex-matched healthy subjects of the same ethnicity and with no history of CTD, asthma or pulmonary history were included as controls. They did not take glucocorticoids or other immunosuppressive medications. The controls were recruited from the medical staff in the Bach Mai hospital and from friends of the patients.

### Measurements of intima–media thickness and plaque frequency of carotid artery

US examinations of all SSc patients and controls were performed at the Radiology department, Bach Mai hospital. B-Mode ultrasonography was used to measure the common carotid IMT. A Phillips iE33 US device with a 7.5–12 MHz linear array transducer was used for measuring IMT in all patients and controls. The commercially available semi-automatic IMT measurement software Math v.3.2.0 was used for carotid IMT measurements. With the subjects in the supine position, the left common carotid artery wall segment was scanned from an anterolateral transducer position and recorded on s-VHS tape. The operator identified the area to measure: a continuous 1 cm segment of the distal 2 cm in the common carotid artery far wall. The software automatically detected the intima–lumen and the media–adventitia interfaces and calculated average carotid IMT (carotid IMT mean), maximum carotid IMT (carotid IMTmax) and the local variation of carotid IMT in the segment (carotid IMTsd). We screened the arteries for carotid plaques in common, internal and external carotids. According to the Mannhein cIMT consensus, a plaque was defined as a focal structure that encroaches into the arterial lumen of ≥0.5 mm or 50% of surrounding IMT value or demonstrates a focal thickening >1.5 mm as measured from the media–adventitia interface to the intima–lumen interface [13].

### Assessment of traditional cardiovascular risk factors for atherosclerosis

Data were obtained from all subjects with respect to previous CVD events, which comprised heart failure, coronary heart disease, stroke and peripheral arterial disease. Heart failure and stroke were defined by physician diagnosis. Coronary heart disease was defined as myocardial infarction or coronary revascularization. Peripheral artery disease was defined by the physician based on clinical manifestations.

The assessed traditional risk factors for atherosclerosis, based on physical examination, were age, sex, BMI, arterial hypertension, diabetes mellitus, smoking (current or former and pack-years), dyslipidaemia and a family history of cardiovascular disease. Obesity was defined as BMI ≥25 kg/m^2^. Blood pressure levels were evaluated as the mean of left and right brachial artery measurements after ≥5 min of rest. Hypertension was defined by blood pressure >140/90 mmHg or use of antihypertensive drugs. In cases when a calcium channel blocker was prescribed for secondary RP, this was not regarded as antihypertensive drug use, except when arterial hypertension was documented previously and the calcium antagonist was given to treat hypertension and RP at the same time. Values of ESR and CRP level were recorded at the time of investigation.

The lipid profile [total cholesterol, high-density lipoprotein (HDL), low-density lipoprotein (LDL) and triglycerides] was measured by routine techniques in the fasting state. Dyslipidaemia was defined by elevated plasma cholesterol (>200 mg/dl for age 18–29 years; >220 mg/dl for age 30–40 years; >240 mg/dl for age >40 years), plasma LDL cholesterol (>160 mg/dl) and/or plasma triglyceride (>200 mg/dl) levels, or use of lipid-lowering drugs, such as statins [14]. Impaired fasting glucose (defined as fasting plasma glucose 100–125 mg/dl) was regarded as prediabetes [15]. All participants were also evaluated for the presence of metabolic syndrome, which was defined according to the National Cholesterol Education Program's Adult Treatment Panel III definition [16].

### Assessment of potential non-traditional and disease-related determinants for atherosclerosis

We evaluated the following non-traditional and disease-related determinants as potential risk factors associated with accelerated atherosclerosis. The extent of skin involvement was evaluated by using the modified Rodnan skin score (MRSS) [17]. Organ involvement was recorded, including RP, pulmonary hypertension, interstitial lung disease, coronary heart disease and peripheral arterial vascular disease (defined by obstructive vessel disease with intermittent claudication), presence of digital pitting scars, digital tip ulcers or gangrene. Interstitial lung disease was defined as the presence of either chest radiographic abnormalities indicative of fibrosis and pulmonary function tests [forced expiratory volume in the first second (FEV1)  < 80%, and forced vital capacity (FVC) < 80% (predicted) and total lung capacity (TLC) <80% and/or >80% predicted FEV1/FVC] or abnormal findings on high resolution computed tomography (HRCT) scan, showing at least one of the following features (with or without restrictive physiological findings on pulmonary function tests): reticulation and fibrosis, traction bronchiectasis, honeycombing and ground-glass opacification [18]. Pulmonary hypertension was defined as an elevated right ventricular systolic pressure (>30 mmHg) by Doppler echocardiography. Articular involvement was determined by clinical evidence of joint swelling, deformity, contractures and tendon friction rubs or radiographic evidence of joint space narrowing or erosion. We also assessed the family history of CTD, disease duration since diagnosis and age at diagnosis.

Previous and ongoing immunosuppressive treatment was recorded, such as use of prednisolone (duration of treatment and mean daily dose), HCQ, AZA and CYC (duration of treatment and cumulative dose, if applicable).

### Autoantibody analysis

Blood samples taken at time of investigation were analysed by standard immuno-fluorescence assay using Hep2 cells for ANA. Specific autoantibodies were tested by ELISA (Euroimmune, Lubeck, Germany) for the following auto-antibodies: anti-topoisomerase 1 (Scl-70), ACA and anti-nuclear RNP autoantibodies.

### Ethics

This study complies with the Declaration of Helsinki and was approved by the local ethics committee at Hanoi Medical University (4020/IRB-HMU). Written informed consent was obtained from each participant.

### Statistical analysis

Values are expressed as the mean (s.d.) when variables were normally distributed and as the median with interquartile range (IQR) in the event of a non-normal distribution. Given that most patients were classified as dcSSc, subset analysis could not be performed. Differences between patients and controls were assessed by Student’s unpaired *t* test, the Mann–Whitney *U* test and χ^2^ test (Pearson χ^2^ or Fisher exact test) as appropriate. Linear regression was used to assess the relationship between IMT and the different groups (SSc patients and healthy controls). An unadjusted analysis, in which no corrections were made for possible confounders, and an adjusted analysis, in which corrections were made for possible confounders, are presented. The level of significance for the association between group and outcome variable was set at a *P*-value of <0.05.

The variables age, gender, BMI and smoking were also studied as potential effect modifiers in the relationship of interest. The correlation between maximum IMT and disease-related factors was assessed by the Spearman rank correlation coefficient because maximum IMT was non-normally distributed. All analyses were carried out with the Statistical Package of Social Science, v.12.1 for Windows (SPSS).

## Results

### Characteristics of patients and controls

Disease characteristics are presented in Table 1. Patients with SSc had a median disease duration of 5 years, ranging from 2 months to 20 years, and had experienced RP for almost 6 years. dcSSC was present in 95.6% of patients and lcSSc in 4.4% of patients. Eighty-three per cent were ANA positive (Table 1). Patients with SSc had significantly higher CRP levels than controls (1.5 *vs* 0.2 mg/dl; *P* = 0.007). Regarding therapy, 89.1% of patients were current users of prednisolone, with a mean cumulative dose of 5.9 (5.7) g for a mean duration of 4.03 (4.02) years, and 70% of patients were treated with an immune-modulating drug (Table 1).

**Table 1:** Disease characteristics of patients with SSc in this study

Disease characteristic	*n* = 46
MRSS, mean (s.d.)	21.3 (8.8)
Interstitial lung disease, *n* (%)	37 (80.4)
Pulmonary artery hypertension, *n* (%)	23 (50.0)
Digital ulcer, *n* (%)	6 (13.0)
Arthritis, *n* (%)	25 (54.3)
Antibody, *n* (%)	
Scl-70 (topoisomerase 1)	19 (41.3)
ACA	1 (2.2)
Nuclear RNP	14 (30.4)
ANA	38 (82.6)
None	8 (17.4)
Prednisolone use, *n* (%)	
None	5 (10.9)
Former	0 (0.0)
Current	41 (89.1)
Cumulative prednisolone dose, mean (s.d.), g	5.9 (5.7)
Immunosuppressive agents, *n* (%)	
Never used	14 (30.4)
Former or current users	32 (69.6)
MTX	12
CYC	12
AZA	6
CSA	0
MMF	2

MRSS: modified Rodnan skin score.

### Cardiovascular events and traditional cardiovascular risk factors

Ten patients had a history of macrovascular events compared with none in the control group (Table 2). Demographic data and traditional risk factors of SSc patients and healthy controls are presented in Table 2. Patients with SSc had significantly higher levels of total cholesterol and lower levels of HDL compared with the age- and sex-matched controls. The use of statins was not different between patients and controls. Patients with SSc had lower BMI than controls. No significant differences were found in other traditional cardiovascular risk factors between the two groups.

**Table 2:** Demographic data and traditional risk factors for cardiovascular disease in SSc patients and healthy controls

Characteristic	Patients (*n* = 46)	Controls (*n* = 42)	*P*-value
Age, mean (s.d.), years	54.8 (11.8)	49.3 (14.7)	0.075
Female gender, *n* (%)	33 (71.7)	35 (83.3)	0.245
BMI, mean (s.d.), kg/m²	19.1 (2.5)	20.5 (2.1)	**0.0133**
Obesity, *n* (%)	1 (2.2)	0	0.4165
Smoking, *n* (%)	5 (10.3)	3 (7.1)	0.5921
Diabetes mellitus, *n* (%)	0	0	–
Pulmonary hypertension, *n* (%)	23 (50)	0	**<0.0001**
Hypertension treated with antihypertensive agents, *n* (%)	4 (8.7)	3 (7.1)	0.7538
Blood pressure, median (IQR), mmHg			
Systolic	115 (90–160)	113 (100–130)	
Diastolic	73 (50–100)	72 (65–80)	
Cholesterol, mean (s.d.), mmol/l			
Total	4.99 (1.15)	3.92 (0.77)	**0.03**
Low-density lipoprotein	2.73 (0.94)	2.06 (0.33)	0.12
High-density lipoprotein	0.92 (0.29)	1.41 (0.59)	**0.01**
Triglyceride	1.08 (0.98)	2.02 (1.02)	0.92
Lipid-lowering drugs, *n* (%)	2 (4.4)	0	0.2473
Family history of cardiovascular disease, *n* (%)	6 (13)	3 (7.1)	0.3846
Cardiovascular events, *n* (%)	10 (21.7)	0	**0.0065**
Coronary heart disease	4		
Cerebrovascular disease	0	–	–
Peripheral arterial vascular disease	4	–	–
Heart failure	2		
CRP, mean (s.d.), mg/dl	1.5 (2.9)	0.23 (0.21)	0.007

Bold text means the difference between the SSc patients and the controls was statistically significant with *P*-value < 0.05. IQR: interquartile range.

### Intima–media thickness and frequency of plaques

The median values for the mean IMT measurements in the common carotid artery were significantly higher in SSc patients: 0.61 mm (IQR 0.42–0.96 mm) compared with the controls 0.47 mm (IQR 0.33–0.61 mm) (*P* < 0.001). We found plaques in 13 (28.3%) of our patients with SSc but in none of the controls (Table 3). There was no difference in IMT or frequency of plaques between patients with or without a previous CVD event.

**Table 3:** Comparison of intima–media thickness and frequency of carotid atherosclerotic plaques between patients with SSc and healthy controls

Parameter	Patients (*n* = 46)	Controls (*n* = 42)	*P* value
IMT, mean (s.d.), mm	0.61 (0.12)	0.47 (0.07)	**<0.01**
Plaque, *n* (%)	13 (28.3)	0 (0.0)	**0.0002**

Bold text means the difference between the SSc patients and the controls was statistically significant with *P*-value < 0.05. IMT: intima–media thickness.

### Correlations of traditional cardiovascular risk factors and disease-related factors with intima–media thickness

Correlations between common carotid artery IMT and traditional or non-traditional risk factors for atherosclerosis are presented in Table 4. Among the SSc patients, there were modest significant positive correlations between IMT and age (*r* = 0.62, *P* < 0.001) and low correlations with disease duration (*r* = 0.37, *P* = 0.01), total cholesterol (*r* = 0.29, *P* = 0.04) and LDL levels (*r* = 0.31, *P* = 0.03) but not with other variables, including autoantibody profile. Also in the healthy controls there was a positive correlation between IMT and age (*r* = 0.47, *P* < 0.001) but with no other variables.

**Table 4:** Correlations of carotid artery intima–media thickness with traditional and non-traditional risk factors for atherosclerosis

Risk factor	Mean carotid IMT of patients (*n* = 46)	
	**Correlation**	***P*-value**
Age, years	*r* = 0.62	<0.001
Disease duration, months	*r* = 0.37	0.01
BMI, kg/m^2^	*r* = −0.1	0.49
Systolic blood pressure, mmHg	*r* = 0.06	0.71
Diastolic blood pressure, mmHg	*r* = −0.07	0.66
Total cholesterol levels, mmol/l	*r* = 0.29	0.04
Triglyceride levels, mmol/l	*r* = 0.03	0.85
HDL cholesterol, mmol/l	*r* = 0.02	0.88
LDL cholesterol, mmol/l	*r* = 0.31	0.03
Glucose levels, mmol/l	*r* = 0.24	0.11
CRP, mg/dl	*r* = 0.19	0.21
MRSS, units	*r* = 0.004	0.98
Pulmonary hypertension, mmHg	*r* = 0.25	0.25

HDL: high-density lipoprotein; IMT: intima–media thickness; LDL: low-density lipoprotein; MRSS: modified Rodnan skin score.

### Associations of traditional cardiovascular and disease-related risk factors with presence of atherosclerotic plaques in SSc patients

In the SSc patients with plaques, mean MRSS and mean IMT were significantly higher compared with patients without plaques (Table 5). Age, disease duration, presence of dyslipidaemia, interstitial lung disease, pulmonary hypertension, glucocorticoid and other immunosuppressive treatments were not different in SSc patients with plaques compared with patients without plaques.

**Table 5:** Exploratory analysis of potential risk factors in patients with or without atherosclerotic plaques

Characteristic	Patients (*n* = 46)		*P*-value
	With plaque (*n* = 13)	Without plaque (*n* = 33)	
Age, mean (s.d.), years	59.9 (11.1)	52.8 (11.1)	0.06
Female sex, *n* (%)	10 (76.9)	23 (69.7)	0.7
Disease duration, mean (s.d.), years	6.4 (7.4)	3.7 (3.3)	0.08
Postmenopausal status, women only, *n* (%)	8 (61.5)	15 (45.5)	0.5
dcSSc, *n* (%)	13 (100)	31 (93.9)	0.0921
MRSS, mean (s.d.)	25.6 (10.9)	19.6 (7.2)	**0.03**
Hypertension, *n* (%)	1 (7.7)	3 (9.1)	0.4
Smoking, *n* (%)	3 (23.1)	2 (6.1)	0.5
Dyslipidaemia, *n* (%)	6 (46.2)	9 (27.3)	0.18
Interstitial lung disease, *n* (%)	11 (84.6)	26 (78.8)	0.5
Pulmonary hypertension, *n* (%)	7 (53.8)	16 (48.5)	0.2
Digital ulcer, *n* (%)	3 (23.1)	3 (9.1)	0.3
Arthritis, *n* (%)	7 (53.8)	18 (54.5)	0.6
ESR above upper reference value, *n* (%)	12 (92.7)	25 (75.7)	0.3
CRP above upper reference value, *n* (%)	11 (84.6)	30 (90.9)	0.4
Total cholesterol, mean (s.d.), mmol/l	5.02 (1.2)	4.9 (1.1)	0.9
Triglyceride, mean (s.d.), mmol/l	1.6 (0.7)	2.2 (1.0)	**0.06**
LDL cholesterol, mean (s.d.), mmol/l	2.8 (0.9)	2.7 (0.9)	0.7
HDL cholesterol, mean (s.d.), mmol/l	1.4 (0.6)	1.4 (0.5)	0.9
Immunosuppressive treatment, *n* (%)	8 (61.5)	24 (72.7)	0.4
Cumulative glucocorticoid dose, mean (s.d.), g	4.6 (4.5)	6.4 (6)	0.3
IMT, mean (s.d.), mm	0.69 (0.16)	0.58 (0.09)	**0.004**

Bold text means the difference between the SSc patients and the controls was statistically significant with *P*-value < 0.05. HDL: high-density lipoprotein; IMT: intima–media thickness; LDL: low-density lipoprotein; MRSS: modified Rodnan skin score.

## Discussion

In this cross-sectional case–control study mainly focusing on patients with SSc of diffuse phenotype, we found a higher frequency of CVD events compared with sex- and age-matched controls. We also found higher IMT values in the common carotid artery and a higher prevalence of carotid plaques in patients with SSc compared with healthy controls, suggesting an accelerated atherosclerosis in these patients that could contribute to the higher risk of cardiovascular disease.

Although macrovascular disease has not typically been regarded as a significant systemic feature in SSc, myocardial infarction and stroke are more common in patients with SSc than in healthy controls [19]. This observation was confirmed in our small study including patients with SSc mainly of the diffuse phenotype. The IMT of the carotid artery is a commonly used marker for subclinical atherosclerosis. A meta-analysis of longitudinal studies including a general population found that for an increase of 0.1 mm in carotid IMT, the age- and gender-adjusted relative risk for myocardial infarction was 1.15 (CI% 1.12–1.17) and the relative risk of stroke was 1.18 (95% CI 1.16–1.21) [20]. Previous studies in patients with SSc showed a marked heterogeneity in IMT results, raising the question of whether IMT measurement is a useful measure to predict CVD in this disease [21, 22]. We found increased carotid IMT compared with healthy controls, and the IMT was associated with longer SSc disease duration, similar to what has been observed in patients with RA [23]. A similar association between IMT and disease duration was also reported in a meta-analysis of carotid IMT in SSc, whereby every 10 year increase in SSc disease duration was associated with an increase in carotid IMT of 0.16 mm [24]. Usually, the modification of IMT at the level of the carotid artery is associated with cardiovascular risk factors or overt cardiovascular disease and atherosclerosis in other vascular beds, although we did not find a difference in IMT or frequency of plaques between patients with or without a previous CVD event.

Carotid IMT and carotid plaques as measured by high-resolution US are well-validated markers of subclinical atherosclerosis and are also biomarkers for future coronary heart disease, stroke and death, both in the general population and in patients with inflammatory rheumatic diseases [25]. Atherosclerosis is known to be a dynamic process, in which endothelial dysfunction, inflammation and traditional cardiovascular risk factors play roles concurrently. Increased carotid IMT has been shown to be correlated with traditional cardiovascular risk factors and to predict future vascular events in healthy individuals [26]. Among the established risk factors, hypertension has the greatest effect on CVD risk in Vietnam. A nationally representative sample revealed that 18.4% of the population aged ≥25 years suffer from hypertension, and the prevalence rate of diabetes standardized by age, sex and area of residence is 6% [27, 28]. The prevalence of hypertension and diabetes in Vietnam appears to be lower than estimates reported in other countries of East Asia and the Pacific [27, 29].

Currently, there is limited information about the underlying mechanisms of atherosclerosis in SSc. Traditional risk factors, such as hypertension, dyslipidaemia, diabetes mellitus, obesity and smoking, are typically not more prevalent in SSc patients than in healthy controls, and this was also the case in our study [30, 31]. We found that a similar distribution of traditional cardiovascular risk factors, such as obesity, diabetes mellitus, hypertension and smoking between SSc patients and controls. Smoking in Vietnam, as elsewhere in Asia, is strongly sex linked. The national prevalence survey found that 47.4% of men but only 1.4% of women used tobacco regularly [32]. In the present study, the very low prevalence of smoking in the SSc cohort and controls, because of a high percentage of women in both groups (71.7% and 83.3%, respectively), is similar to what has been reported in other Vietnamese SSc cohorts [7]. The background traditional risk of CVD in Vietnam is relatively low. The routine measurement of IMT of the carotid arteries to identify atherosclerotic lesions is useful in detecting SSc patients at a high risk for cardiovascular events, especially in Vietnamese patients. Every year, intensive training courses on vascular US are organized by cardiovascular institutes and radiology centres at major hospitals in Vietnam to improve the US skills of radiologists and cardiologists.

Hyperactivation of the immune system and systemic inflammation can lead to premature atherosclerosis and early occurrence of its clinical manifestations [33]. Myocardial microvasculopathy and focal and diffuse myocardial fibrosis are suggestive of primary heart involvement in SSc. Myocardial fibrosis has been postulated to be the consequence of repeated focal ischaemia owing to microvasculopathy. A silent inflammatory process could be the substrate for the development of myocardial fibrosis [34]. These findings support the association of myocardial fibrosis with disease severity and suggest a likely concurrent fibrotic process affecting both the skin and the myocardium. The development of accelerated atherosclerosis in SSc has been proposed to be influenced by viral agents, such as herpes viruses, or by *Chlamydiapneumoniae* or *Helicobacterpylori*, in addition to immune reactions including anti-endothelial cell antibodies, or ischaemia–reperfusion injury [35]. Increased levels of CRP, homocysteine, von Willebrand factor and vascular adhesion molecules, all of which are associated with the atherosclerotic process, have been reported in patients with SSc, whereas data on serum levels of lipids are conflicting [9, 36]. In our population, CRP levels, although within normal limits, were significantly elevated compared with the healthy controls, although CRP levels were not associated with IMT or the presence of carotid plaque. We also found a higher prevalence of dyslipidaemia in patients with SSc than in controls. This is in line with a previous report of elevated lipoprotein(a) in patients with SSc compared with healthy controls, although no significant difference was found in other cholesterol parameters or triglycerides [37]. There is some evidence that novel markers of atherosclerotic risk such as homocysteine, lipoprotein and oxidized LDL are more prevalent in SSc, but these results have not been substantiated in more extensive studies and were not measured in our cohort [38, 39].

Our observed frequency of atherosclerotic plaques is lower (28%) compared with studies of larger patient cohorts of patients with SSc [40, 41]. The explanation for this difference is not clear, but remarkably, none of the controls had any plaques in our study, which might be explained by a lower age of the patients in our study compared with previous reports [40, 42]. We found that SSc patients with plaques had higher mean MRSS in comparison to patients without plaques. Cheng *et al.* noted previously that the carotid wall of patients with SSc was stiffer than that of controls, and in subgroup analysis stiffness was more pronounced in dcSSc than in limited cutaneous scleroderma, which could not be confirmed in another study [21, 43]. Different disease characteristics, such as disease duration, ACA positivity, cumulative glucocorticoid dose and MRSS (negatively) have been reported to be associated with cardiovascular disease and atherosclerosis in patients with SSc [22, 41, 44]. Subclinical atherosclerosis plaque occurrence in SSc patients was reported to be associated with smoking, higher blood pressure, impaired kidney function, high levels of IL-6, serum levels of vascular cell adhesion molecule 1 and with ACA [22]. However, this could not be confirmed in our study, perhaps because of the low number of cases or different phenotypes of SSc, given that our cohort mainly included dcSSc. Measurement of skin thickness is used as a surrogate for disease activity, severity and mortality in patients with SSc. The gold standard for measuring skin fibrosis in SSc is the MRSS. It is essential for the Vietnamese physicians caring for patients with SSc to perform the MRSS in clinical practice. Most rheumatologists receive MRSS training through the Vietnamese Rheumatology Association, workshops and mentor teaching to improve technique.

Treatment with immunosuppressive drugs, especially CSs, influences the atherogenic process. Glucocorticoids are considered to have atherogenic properties, like AZA, whereas HCQ and MTX might have protective effects against atherosclerosis [45, 46]. In general, it is difficult to establish whether the observed associations between immunosuppressive drugs and atherosclerosis are attributable to the immunosuppressive drugs themselves or to their effect on the disease activity of the autoimmune disease. In our study, 89% of SSc patients received glucocorticoid at the time of inclusion, and 70% of patients were former or current users of immunosuppressive drugs. However, we found no association between IMT and cumulative prednisolone dose or use of other immunosuppressive drugs. Glucocorticoids are mainly prescribed to Vietnamese patients with dcSSc or overlap or with internal organ involvement. The great majority of patients were prescribed with low to medium doses, ≤15 mg/day, mainly in combination with other immunosuppressants or monotherapy. There are fewer SSc patients undergoing treatment with MMF, AZA, CSA and rituximab because these drugs are not covered by health insurance, limiting their prescription in Vietnam.

There are limitations of our study. Although it is a small cohort, it is one of the largest sample-sized case–control studies evaluating carotid IMT and carotid plaques in Vietnamese patients with SSc. Another limitation is the selection of patients mainly of the dcSSc phenotype and a cross-sectional design. Our study was conducted in Bach Mai hospital, which is a general hospital at the central level with mainly treatment for severe patients in northern Vietnam. The SSc patients with the diffuse subtype generally have more severe disease. The onset of internal symptoms in patients with lcSSc tends to occur later and less frequently compared with dcSSc. The high prevalence of patients with dcSSc in our study is probably a selection bias, because patients with lcSSc do not come to our hospital. However, no significant difference in carotid IMT was reported between disease subsets in previous reports [47, 48]. Another limitation is that we did not include measures of endothelial function, such as flow-mediated dilatation. Moreover, measurements of IMT were limited to the common carotid artery and not extended to other segments of the arteries. The prevalence of increased IMT and/or plaques is substantially higher in the carotid bulb or internal carotid artery in the general population; therefore, for this intended quantitative comparison between SSc patients and controls we found it more appropriate to exclude the carotid bulb or internal carotid artery in our measurement, which might have resulted in a higher value of IMT in patients.

In conclusion, our study supports previous reports that patients with SSc, in particular the subgroup of dcSSc, are at high risk of cardiovascular events, compared with age- and sex-matched controls. This could be explained by an accelerated atherosclerosis, measured as a higher IMT in the common carotid artery, and a higher prevalence of carotid plaque observed when compared with age-matched controls, both indicating subclinical atherosclerosis. Furthermore, the common carotid artery IMT was associated with traditional risk factors for cardiovascular disease, such as age and dyslipidaemia, whereas the plaque frequency was associated with a more severe skin disease. Considering the high frequency of atherosclerosis in SSc and the burden of cardiovascular disease in mortality of SSc, assessment of cardiovascular risk factors and prevention of cardiovascular disease represent an opportunity to reduce morbidity and mortality in SSc. Further research into the mechanism of increased risk of atherosclerosis in SSc is warranted.
